# Intersectional Challenges of ADL and Financial limitations: The Mediating Role of Participation in WellBeing

**DOI:** 10.1093/geroni/igaf122.3070

**Published:** 2025-12-31

**Authors:** Qiwei Li, Xiaoli Li, Cheng Yin

**Affiliations:** California State University, Fresno, Fresno, California, United States; Southern Illinois University, Carbondale, Illinois, United States; University of North Texas, Denton, Texas, United States

## Abstract

**Background:**

Older adults with both ADL limitations and financial constraints face significant challenges in achieving well-being. While community participation was reported as a factor to improved well-being, little is known about its mediating role in this population facing the intersectional difficulties brought by ADL and financial limitations. This study applies Structural Equation Modeling (SEM) to examine the relationships between ADL, financial constraints, participation, and well-being.

**Methods:**

Using waves 5 to 9 of NHATS data (n = 4,152), we specified latent constructs for Well-being, Participation, ADL, and Financial Constraints within an SEM model. Mediation effects were assessed by decomposing direct and indirect effects and calculating the mediated proportion. Covariates were included in the model as well.

**Results:**

The model demonstrated good fit. ADL had a direct negative association with well-being (β = -0.423, p < .001) and participation (β = -0.475, p < .001). Participation positively predicted well-being (β = 0.255, p < .001), indicating mediation. Financial constraints negatively impacted participation (β = -0.075, p < .001) and well-being (β = -0.083, p < .001). Marital status (β = 0.041, p = .008) and race (β = 0.035, p = .027) were also significant well-being predictors. Participation mediated 22.3% of the ADL to well-being relationship and 18.8% of the financial constraints to well-being relationship.

**Conclusion:**

Participation serves as a key mediator between ADL limitations, financial constraints, and well-being. These findings highlight the importance of social engagement interventions to support well-being among older adults facing both functional and financial barriers.

